# Immune Selection and Within-Host Competition Can Structure the Repertoire of Variant Surface Antigens in *Plasmodium falciparum* - A Mathematical Model

**DOI:** 10.1371/journal.pone.0009778

**Published:** 2010-03-22

**Authors:** Sander P. van Noort, Marta C. Nunes, Gareth D. Weedall, Lars Hviid, M. Gabriela M. Gomes

**Affiliations:** 1 Instituto Gulbenkian de Ciência, Oeiras, Portugal; 2 Respiratory and Meningeal Pathogens Research Unit, University of the Witwatersrand, Johannesburg, South Africa; 3 Centre for Medical Parasitology at University of Copenhagen and Rigshospitalet, Copenhagen, Denmark; Walter and Eliza Hall Institute of Medical Research, Australia

## Abstract

**Background:**

The evolutionary mechanisms structuring the expression pattern of variant surface antigen (VSA) families that allow pathogens to evade immune responses and establish chronic and repeated infections pose major challenges to theoretical research. In *Plasmodium falciparum*, the best-studied VSA family is erythrocyte membrane protein 1 (PfEMP1). Each parasite genome encodes about 60 PfEMP1 variants, which are important virulence factors and major targets of host antibody responses. Transcriptional switching is the basis of clonal PfEMP1 variation and immune evasion. A relatively conserved subset of PfEMP1 variants tends to dominate in non-immune patients and in patients with severe malaria, while more diverse subsets relate to uncomplicated infection and higher levels of pre-existing protective immunity.

**Methodology/Principal Findings:**

Here, we use the available molecular and serological evidence regarding VSAs, in particular PfEMP1, to formulate a mathematical model of the evolutionary mechanisms shaping VSA organization and expression patterns. The model integrates the transmission dynamics between hosts and the competitive interactions within hosts, based on the hypothesis that the VSAs can be organized into so-called dominance blocks, which characterize their competitive potential. The model reproduces immunological trends observed in field data, and predicts an evolutionary stable balance between inter-clonally conserved dominance blocks that are highly competitive within-host and diverse blocks that are favoured by immune selection at the population level.

**Conclusions/Significance:**

The application of a monotonic dominance profile to VSAs encoded by a gene family generates two opposing selective forces and, consequently, two distinct clusters of genes emerge in adaptation to naïve and partially immune hosts, respectively.

## Introduction

Although people living in malaria endemic regions typically carry *Plasmodium falciparum* parasites throughout life, clinical symptoms decrease markedly with age [1]. Naturally acquired immunity to the disease involves many components and their relative importance is only partially understood [2]. However, antibodies undoubtedly form a critical component of immunity to the asexual blood stages [3], and the parasite-encoded variant surface antigens (VSAs) exported to the surface of infected erythrocytes (IEs) are important targets [4], [5]. *P. falciparum* parasites possess several VSA families, of which the best characterized is *P. falciparum* erythrocyte membrane protein 1 (PfEMP1) encoded by approximately 60 *var* genes per genome [6]. The level of diversity among *var* genes varies greatly both between and within individual genomes [7]. PfEMP1 variants mediate adhesion of IEs to different host endothelial receptors, and different binding properties have been associated with distinct patterns of sequestration and pathogenesis [8]. The importance of PfEMP1 in malaria pathogenesis has motivated the development of theoretical models of diversity and immune selection [9]–[11].

Individual IEs express only a single PfEMP1 variant at a time [12]. Early in blood stage infection after liver release, many *var* genes are transcribed by the various IEs, but gradually this pattern changes and particular subsets of *var* genes are predominantly expressed [13], [14], while others may still be expressed at low frequency due to transcriptional switching [15], [16]. Variants that predominate in the early phase of infection probably have higher effective multiplication rates (possibly due to more efficient endothelial sequestration rates) or higher on-switching rates. In any case, the history of PfEMP1 expression is recorded in the antibody repertoires that accumulate in individual hosts, regardless of the molecular basis of the sequence of expression [4], [17]–[19].

There is evidence that there is a threshold of PfEMP1 expression necessary for induction of an immune response [20]. If so, low-level or heterogeneous expression of PfEMP1 variants, such as in the early stages of infection, might not be sufficient to induce immunity. As the immune system disables IEs expressing the dominant VSA, the parasite is either cleared from the host or parasites expressing an antigenically distinct VSA will come to dominate the infection [21]. When a VSA is no longer expressed, antibody levels against it decrease, but immunological memory persists and antibody levels can be rapidly restored upon re-exposure [22]–[24].

Here we investigate the role of variation in adhesion properties and cumulative antibody repertoires in selecting for the observed patterns in expression [25], [26]. Integrating these individual-level processes into a mathematical model of *P. falciparum* transmission, we refine the requirements for the emergence of realistic variation in VSA expression at the population level. The model allows for multiple genotype infections, encapsulating a form of within-host competition that gives selective advantage to parasites expressing more dominant VSAs [27].

Although global *var* gene diversity is immense [28], there is increasing evidence that there exist restricted subgroups of antigenically similar VSAs that have a selective advantage in naive hosts and are associated with severe disease (called “high-dominance” VSAs here), whereas other more diverse PfEMP1 variants (called “low-dominance” VSAs) are more common in the uncomplicated and sub-clinical infections of more immune hosts [26], [29]–[35]. Our model suggests that within-host competition selects for a relatively conserved repertoire of high-dominance VSAs, while a diverse repertoire of low-dominance forms is maintained by their ability to remain unrecognised by host immunity for extended periods allowing chronicity of infections. We propose this mechanism of two-level selection as an evolutionary explanation for the subdivision of large VSA families such as PfEMP1.

## Results

### Model outline

On the basis of available experimental evidence summarized above, we hypothesize that the global repertoire pool of variants within a given VSA family can be ordered into a dominance hierarchy that determines the order in which they are expressed in an infection. The dominance hierarchy is considered the aggregated result of a variety of selective factors, including adhesion avidity, receptor availability, metabolic cost, gene switching rates, and immuno-dominance, to name a few. The parasites in an infection will therefore tend to express the most dominant variant to which the host does not have pre-existing immunity. As immunity to the initially dominant variant is acquired, continued parasite survival depends on the ability to switch away from this variant, and switching back to the originally expressed variant will be unsuccessful as long as protective levels of antibody with specificity for this variant persist.

Before constructing a model we must devise a scheme to aggregate the immense VSA diversity in a way that is both biologically meaningful and mathematically tractable. As we are mainly interested here to explore host population-level processes, we define “dominance blocks” as groupings of undefined numbers of consecutive variants. Since dominance reflects preferential expression, it is assumed here that antigenic switching during a single infection occurs among consecutive variants within a dominance block, and is therefore not visible at the scale of blocks. Dominance blocks are thus a convenient unit for the construction of transmission models at this level.

**Figure 1:**
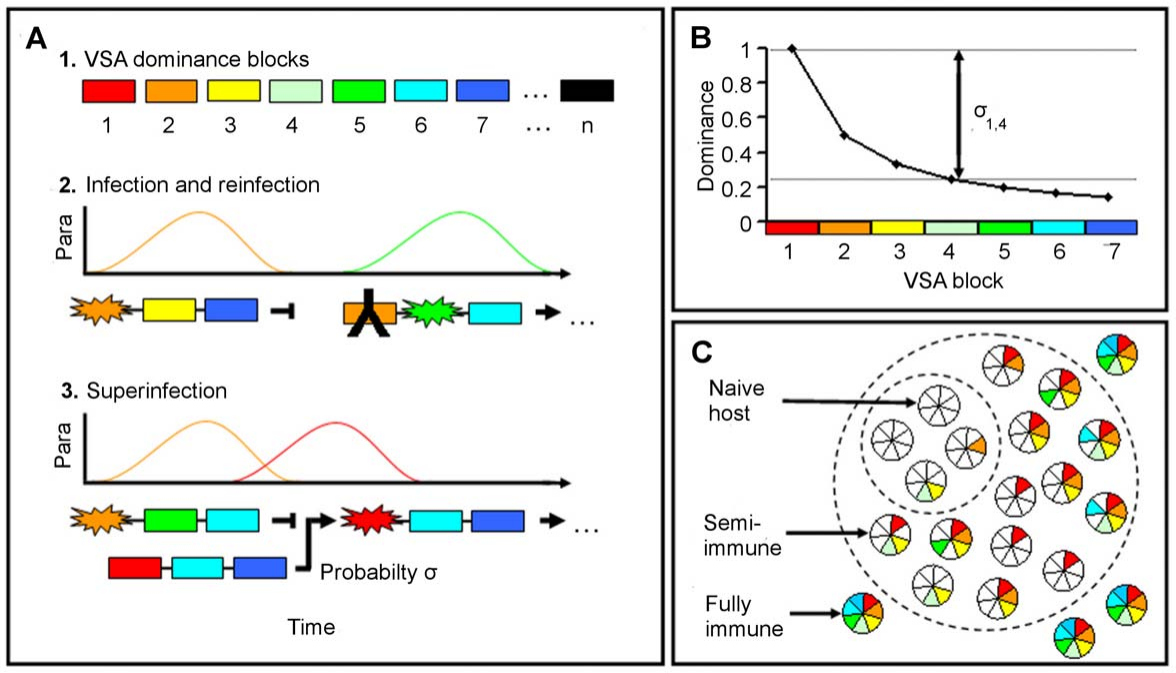
Schematic representation of infection dynamics. (A) VSA variants are organized into dominance blocks, where dominance ranges from the highest (VSA block 1) to the lowest (VSA block n) (A, topl). Each parasite genotype contains VSAs from a fixed number of VSA blocks. In a naive host, a parasite clone (illustrated by a set of 3 VSA blocks) expresses VSAs from the most dominant block. Immunity is illustrated by an antibody and expression by a star. The host mounts an immune response to the expressed VSAs and eventually the infection is cleared. On a subsequent infection the host is already immune to VSAs from previously expressed blocks, leading to the expression of VSAs belonging to the next most dominant VSA block (A, middle). When an infected host is exposed to a new parasite which encodes VSAs from a more dominant block, the resident parasite can be replaced with a probability *σ* (A, bottom). (B) Implementation of dominance hierarchy of 7 VSA blocks, such that *σ_1,4_* is the probability of superinfection when VSA block 1 invades a host with a resident parasite expressing VSA block 4. (C) Heterogeneous immune repertoires among hosts. Hosts acquire specific immunity with exposure, represented by colours matching VSA blocks in previous panels. The immune repertoire may contain gaps if a host has not been exposed to a particular variant block. Fewer hosts are susceptible to VSA 1 (small dashed circle) than to VSA 7 (large dashed circle).

Estimates of the duration of a *P. falciparum* infection are in the order of 200 to 700 days [36]–[38], with peaks in parasitaemia every 20–25 days [39]. If peaks in parasitaemia correspond to clonal replacement of one variant by another, and in the absence of substantial cross-reactive immunity among intraclonal variants [40], between eight (an infection that lasts 200 days with peaks every 25 days) and 35 (peaks every 25 days of a 700 day infection) different VSAs can be assumed to be expressed in the course of an untreated monoclonal infection. This collection of VSAs corresponds to a dominance block. The model will be formulated in terms of blocks of VSAs, indexed such that a lower index represents a higher dominance (Fig. 1a, top). Each parasite can thus be assumed to possess between approximately two (at 35 variants per block) and seven (at eight variants per block) dominance blocks of a given VSA family such as the 60-member PfEMP1 family, and should therefore be able to re-infect a given host at least a corresponding number of times (each time expressing variants from a new block) before all family members have been expressed (Fig. 1a, middle). In reality, the number may well be higher, as levels of variant-specific antibodies often decline fairly rapidly once exposure to the variant ceases [22]–[24].

Multiple-clone infections are very common in malaria endemic areas. We hypothesize that VSA dominance not only determines the order of VSA expression of a single clonal lineage, but also the dynamics of multiple-clone infections. When an already infected host is exposed to a new parasite, we assume that the transmission potential of the invader in relation to the resident increases with the dominance difference between the respective VSAs. Others [41] have shown that such weighted processes can be mathematically simplified while maintaining the essence of the model dynamics, by assuming the polarized view that the invader replaces the resident parasite (superinfection) with a probability *σ* (Fig. 1a) and is cleared otherwise. The probability *σ* is formally defined as a function of the difference between the dominance blocks expressed by invader and resident parasites (Fig. 1b).

As hosts in the population gradually acquire immunity to individual dominance blocks, they remain susceptible only to parasites which express blocks of lower dominance (Fig. 1c). A correlation between dominance and disease severity is implicit in the model, and is used to evaluate its performance. Immunity to specific VSA blocks wanes over time. The mathematical formalism of the model is provided in the Methods section.

### Model simulations

We will describe equilibrium results from simulations of model realisations where parasites are described by four dominance blocks drawn from a pool of seven. This greatly simplifies the description of the model output but retains the generality of the model performance.

**Figure 2:**
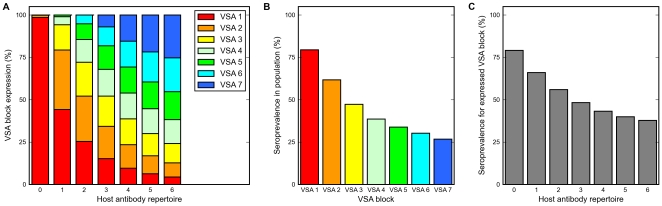
Expression and seroprevalence of VSAs at endemic equilibrium. (A) VSA expression within hosts with different levels of past exposure (measured by host antibody repertoire). (B) Seroprevalence for the different VSA blocks (trend displayed in Fig. 3 of [29]). (C) Seroprevalence for the expressed VSA as a function of host antibody repertoire (trend displayed in Fig. 2c of [42]).

The vast majority of hosts without any pre-existing VSA-specific antibodies is predicted by the model to be infected by parasites expressing VSAs belonging to dominance block 1. As host repertoires of VSA-specific antibodies broaden, the probability that their infections will be dominated be parasites expressing VSAs from dominance blocks lower in the hierarchy increases, and the ability to predict which block is expressed in a given host decreases (Fig. 2A). If we associate high-dominance VSAs with more severe forms of malaria, and note that the antibody repertoire broadens with age, this output fits the observations that overall malaria severity decreases with age in endemic areas [1], and that low immunity and young age are associated with infections dominated by serologically similar VSAs, whereas VSA expression in older, more immune individuals with uncomplicated infections is much more diverse [26], [29]. The host's capacity to clear an infection before exhausting the VSA repertoire of the infecting parasite (see Model outline above) and the non-random sequential expression of variants from high- to low-dominance, furthermore leads the model to predict a population-level gradient from high prevalence of hosts with antibodies against high-dominance VSAs to low prevalence of hosts with antibodies against low-dominance VSAs (Fig. 2B). The model also predicts a negative correlation between the size of the antibody repertoire and the seroprevalence against the expressed VSA, meaning that VSAs expressed in hosts with broad antibody repertoires are less recognized in the host population than VSAs expressed in hosts with narrow antibody repertoires (Fig. 2C). The trends in Fig. 2B,C correspond well with field data [25], [26], [42].

**Figure 3:**
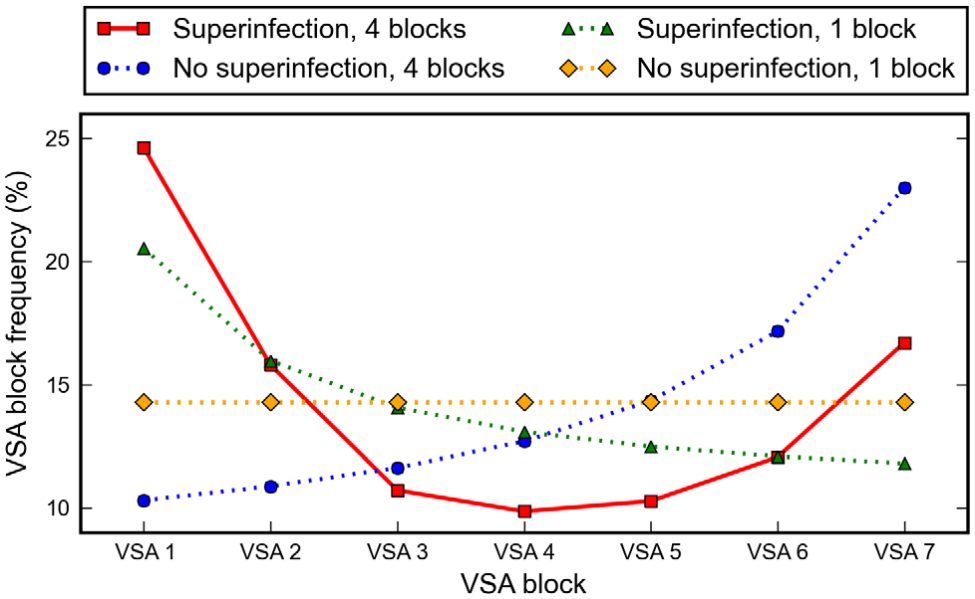
Frequency distributions of VSAs in the parasite population at endemic equilibrium. Opposing selection pressures favour VSA blocks at each end of the dominance hierarchy (solid red line), suggesting a mechanism behind the two clusters displayed in Fig. 3 of [35]. Superinfection selects for high-dominance VSAs (compare solid red line and dotted blue line). When parasites contain multiple VSA blocks, acquired immunity selects for low-dominance variants (compare solid red line and dotted green line). Without superinfection and only a single VSA block per parasite, there is no frequency difference between the different VSA blocks (dotted yellow line).

The model predicts that the high prevalence of antibodies with specificity for dominant VSAs favours parasites encoding low-dominance variants while parasites encoding the high-dominance variants are simultaneously favoured because of their ability to superinfect and displace resident parasites from infected hosts (Fig. 3, solid red line). These opposing selective forces shape the parasite population such that a typical parasite genome will contain VSAs from both high- and low-dominance VSAs, while variants of intermediate dominance will be the least frequent in the parasite population. Indeed, the selection for high-dominance VSAs becomes weaker when the ability to superinfect is removed from the model, while assigning only a single VSA block to each parasite weakens the selection for low-dominance VSAs. Parasite genomes encoding multiple copies of the same VSAs are outcompeted by parasite genomes encoding the maximum number of distinct VSAs, consistent with high inter-locus diversity seen in the genome [6].

**Figure 4:**
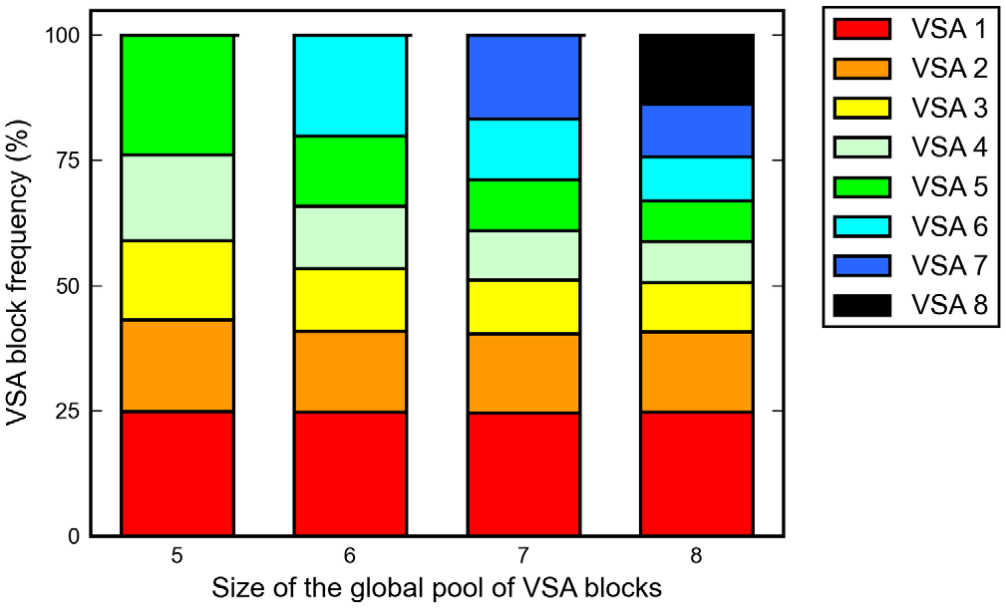
Frequency distributions of VSAs at endemic equilibrium for increasing sizes of the global pool. Increasing the size of the global pool leaves the frequency of high-dominance VSAs unchanged, and diversity accumulates among the low-dominance VSAs. The frequency distribution for a global pool of 7 blocks is also shown in Fig. 3 (solid red line).

Finally, we explore the dependence of VSA distributions on the size of the available pool of variants. By increasing the size of the VSA pool from 5 to 8 blocks, the model predicts that the prevalence of high-dominance VSAs is essentially independent on the global size of the pool, while low dominance VSAs tend to become more heterogeneously distributed throughout the parasite population (Fig. 4). In reality, the number of antigenically distinct VSAs, and thereby the number of possible dominance blocks, is likely to be very large, leading to a restricted set of highly dominant and serologically similar VSAs, and a much larger set of serologically diverse VSAs each with low prevalence.

## Discussion

Immunity to malaria following natural exposure to *P. falciparum* is developed over several to many years, and sterile immunity is probably never achieved. However, immunity to severe disease develops much faster than protection from uncomplicated disease and asymptomatic parasitaemia [43]. It has been suggested that this epidemiological pattern is due to the importance of VSA-specific immunity for clinical protection, to the non-random order in which immunity to specific VSAs is acquired, and to the association between particular VSAs with specific disease syndromes [5], [44].

We present a mathematical model which implements the interplay between two opposing selection pressures; one that favours virulent (high-dominance) VSAs in non-immune hosts, the other facilitating non-virulent (low-dominance) VSAs that allow chronic infections in individuals with substantial VSA-specific immunity. Our results complement previous models that have addressed expression patterns [10], [45] and acquisition of immunity [46].

We introduce the concept of dominance blocks to describe the competitive interactions among the different VSAs of a single parasite (intra-clonal variation) and among the VSAs of different parasite clones (inter-clonal diversity). The model dynamics of VSA expression, within and between hosts, all follow from the dominance hierarchy. The preferential expression of high-dominance VSAs in naive hosts leads to host immunity and quickly exhaust the pool of susceptible hosts available to them. Low-dominance VSAs, on the other hand, allow persistent infections and thereby ensure efficient transmission from host to host. The ability of parasites encoding high-dominance VSAs to superinfect, favours their conservation. The output of our model is compatible with field observations.

The existence of different levels of immune selection acting on VSAs has been suggested through the application of network approaches to serological data [11]. It is reinforced here with a model that is the first to combine intra-host competition and inter-host transmission to investigate the combined effects of selection at multiple levels. The model suggests a mechanism for the observed structuring of VSA into distinct clusters, reproducing important features of serological observations from the field. Although the results are general, it would be interesting to investigate how they might be modulated by transmission intensity and cross-immunity.

Molecular studies have shown that *var* genes, previously classified into five major groups (A–E), could be organized into two broad clusters [35], [47]. A relatively conserved cluster consists of restricted subsets of structurally related variants transcribed by parasites obtained from individuals with limited or no immunity preferentially transcribe [14], [48], and parasites selected *in vitro* for reactivity of IEs with IgG from children with limited immunity [49]. Transcription of two of these subsets (Group A and Group B/A) has repeatedly been associated with severe disease [30]–[34]. A much more diverse cluster contains Group C which has been largely associated with asymptomatic infections [31].

In summary, we present a model that identifies the mechanisms that might be driving the evolution of separate clusters of VSAs, as seen for the *var* gene subfamilies of *P. falciparum*. The hypothesis predicts a restricted subset of high-dominance VSAs associated with severe malaria, and genetically and immunologically diverse low-dominance VSAs related to uncomplicated and asymptomatic infection.

## Methods

The model, constructed as a system of ordinary differential equations, integrates dynamics at two levels (pathogen competition at the individual level as immunological memory accumulates, and pathogen transmission at the population level) in a form that is inevitably dense. In the interest of clarity we construct the model in a stepwise manner.

### Parasites with a single dominance block

We write a first version where each parasite in characterised by only one dominance block, and then generalise for multiple blocks. Consider a pathogen population comprising a diversity of VSA blocks, indexed by the set 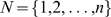, ordered by inverse dominance. Hosts are classified into uninfected (*S*) or infected (*I*) and by the subset of blocks to which they have immunological memory (*h*). Infected hosts are further classified by the blocks that they are currently infected with (*p*). This system is written as
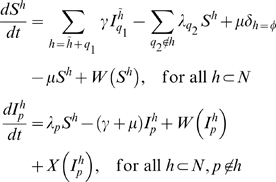
(1)where *q*_1_ indicates the VSA block by which the host was previously infected while having immunological memory 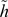, *q*_2_ indicates the VSA block by which the susceptible host will be infected, *γ* is the rate of recovery from infection, *μ* is the rate of birth and death, 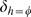 is a delta function indicating that individuals have no immunity at birth, 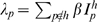 is the force of infection of variant *p*, and *W* and *X* are functions that determine waning immunity (2) and superinfection (3), respectively. For simplicity of notation, we write 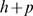, instead of 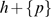, even though *h* is a set and *p* is an element.

Waning immunity is implemented as
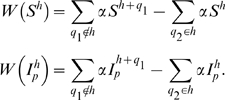
(2)where *q*_1_ indicates the VSA block for which the host previously had immunity, and *q*_2_ indicates the VSA block for which the host is losing immunity.

Superinfection is implemented such that hosts currently infected by a block, *p*, can be superinfected by a higher dominance block, *q*_2_, with a force of infection 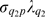. By superinfection, we mean that hosts become infectious with the new block while the old variant is cleared and added to the repertoire of immunological memory. This is formalised as
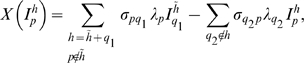
(3)where *q_1_* indicates the VSA block by which an infected host was previously infected while having immunological memory 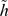. The coefficient 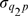 (and equivalently 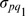) is defined such that the rate of superinfection increases with the difference in dominance between blocks (see Figure 1b)
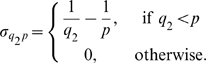
(4)


### Parasites with multiple dominance blocks

The system is readily generalisable to a scheme where each parasite is characterised by multiple VSA blocks. Consider a parasite, 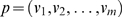, characterized by *m* blocks drawn from a pool 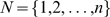 and ordered by inverse dominance. Upon infection by a parasite, *p*, a host with immunological memory, *h*, will express the most dominant block for which the host does not have immunity, 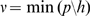. The system is written as
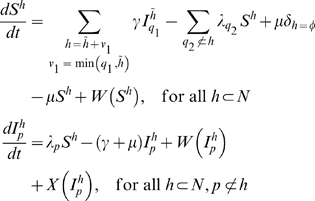
(5)where *q*_1_ indicates a parasite expressing VSA block *ν*_1_ by which the susceptible host was previously infected while having immunological memory 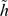, *q*_2_ indicates the parasite by which a susceptible host will be infected, and 
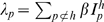
 is the force of infection of parasite *p*. Waning immunity is implemented as
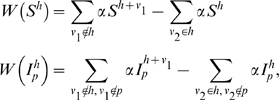
(6)where *ν*_1_ indicates the VSA block for which the host previously had immunity, and *ν*_2_ indicates the VSA block for which the host is losing immunity.

Superinfection is determined by
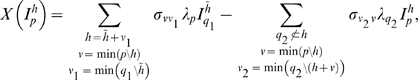
(7)where *q_1_* indicates the parasite expressing VSA block *ν*_1_ by which the host was previously infected while having immunological memory 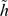, and *q_2_* indicates the parasite expressing VSA block *ν*_2_ by which the host will be superinfected, The coefficients *σ* are as in (4).

The principal steps in this process are represented diagrammatically in the Supporting Information (Figure S1). The parameters describing the rates of transition between compartments take values in accordance with previous studies (Aguas et al 2008): birth and death (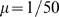); recovery from infection (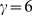); loss of immunity (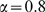); transmission (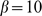). The time unit is one year.

## Supporting Information

Figure S1 Model diagram
(0.09 MB PDF)
