# Supplementary material for: Immune Selection and Within-Host Competition Can Structure the Repertoire of Variant Surface Antigens in Plasmodium falciparum - A Mathematical Model
Source: PLoS One. 2010 Mar 22;5(3):e9778. doi: 10.1371/journal.pone.0009778 (PMC2842302; doi:10.1371/journal.pone.0009778)

Figure S1 - Model diagram

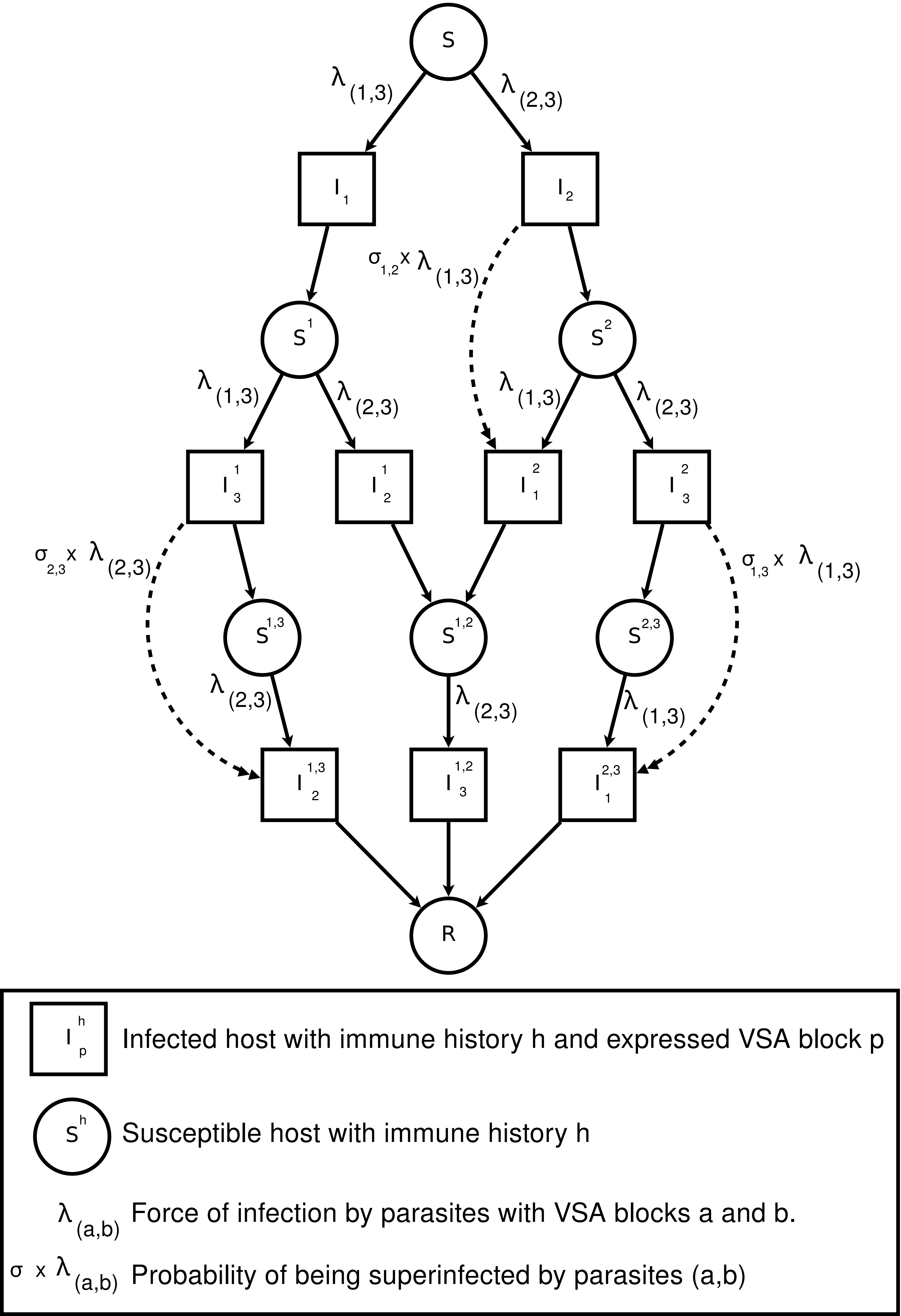

Supplement: Figure S1 — Model diagram (0.09 MB PDF) [file pone.0009778.s001.pdf]
